# Microglia in Health and Disease: A Unique Immune Cell Population

**DOI:** 10.3389/fimmu.2018.01779

**Published:** 2018-08-15

**Authors:** Alessandro Michelucci, Michel Mittelbronn, Diego Gomez-Nicola

**Affiliations:** ^1^NORLUX Neuro-Oncology Laboratory, Department of Oncology, Luxembourg Institute of Health, Luxembourg City, Luxembourg; ^2^Luxembourg Centre for Systems Biomedicine, University of Luxembourg, Esch-sur-Alzette, Luxembourg; ^3^Luxembourg Centre of Neuropathology, Dudelange, Luxembourg; ^4^Laboratoire National de Santé, Dudelange, Luxembourg; ^5^School of Biological Sciences, University of Southampton, Southampton, United Kingdom

**Keywords:** microglia, ontogeny, inflammation, neurodegeneration, brain tumour

**Editorial on the Research Topic**

**Microglia in Health and Disease: A Unique Immune Cell Population**

Microglia are essential for the development and function of the adult brain. Their ontogeny, together with the absence of turnover from the periphery and the singular environment of the central nervous system (CNS), makes microglia a unique cell population compared with other tissue macrophages. Supporting this notion, recent transcriptional studies have revealed that microglia display specific gene expression signatures that are clearly distinct from other brain and peripheral immune cell populations in the healthy and diseased CNS (1).

The unique properties and functions of microglial cells, such as their role in synaptic pruning or the exceptional capacity to scan the brain parenchyma and rapidly react to its perturbations, have emerged in recent years. In the coming years, understanding (i) how microglia acquire and maintain their unique profiles to fulfill distinct tasks in the healthy CNS and (ii) how these are altered in disease, will be essential to develop strategies to diagnose or treat CNS disorders with an immunological component.

In this research topic, four original articles and four reviews cover several aspects of microglial biology, ranging from their origin and the functional role of microglia during development and lifespan, their molecular properties compared with other brain and peripheral immune cells to microglial phenotypes and functional states in neurodegenerative diseases and brain tumors.

In particular, Menassa and Gomez-Nicola describe the various historical schools of thought that had debated the origin of human microglial cells. The data that have recently been accumulated on microglial dynamics in the developing human brain, together with the evidence obtained from rodent studies on the functional role of microglia during development, allowed them to identify limitations for the existing approaches in human studies.

Savage et al. and colleagues focus on defining the distinctive ultrastructural features of microglia and the unique insights into their function that can be provided by electron microscopy. These techniques will further revolutionize the study of microglia across lifespan, brain regions, and contexts of health and disease.

Ayana et al. conducted a meta-analysis of brain cells, using publicly available datasets, for deciphering microglia-specific expression profiles, thus identifying putative novel human microglia markers across lifespan and different brain regions.

In her review, Sevenich summarizes recent developments in evaluating the distinct functions of brain-resident and recruited myeloid cells in neurodegenerative disorders and brain cancers, thus underlying disease- and cell-type-specific effector functions of microglia and macrophages.

Walentynowicz et al. attempt to study glioma-associated microglia/macrophage functional states by identifying marker genes from different experimental models and clinical samples, revealing only a small set of common genes, thus reflecting divergent responses depending on specific clinical and experimental settings.

A study by Blank et al. suggests that early retinal microglia activation represents a first step in the pathological cascade of retinitis pigmentosa, which might initiate or accelerate photoreceptor degeneration, thus pointing out that activated microglial cells may be responsible for the onset or amplification of the degenerative process.

In this line, Zhou et al. show that the severity of retinal degeneration is related to microglia polarization. Specifically, the authors revealed that alpha-1 antitrypsin, a novel immunomodulatory agent in autoimmune diseases and transplantation, shifts microglia toward an anti-inflammatory phenotype along with a protective effect on retinal degeneration.

Finally, Kounatidis and Chtarbanova review the recent findings obtained in the fruit fly (*Drosophila melanogaster*) on the contribution of glial innate immune pathways in lifespan and neurodegeneration. In particular, the NF-κB pathway and the phagocytic ability appear to be major contributors to lifespan, possibly through an immune–neuroendocrine axis.

In conclusion, this research topic provides a comprehensive overview of our current understanding of several cellular and molecular mechanisms that make microglia a unique immune cell population within the healthy CNS as well as under inflammatory, neurodegenerative, and tumorigenic processes. Furthermore, an emerging key aspect of microglia is their heterogeneity across the CNS under homeostatic or pathological conditions. Established methodologies combining single-cell approaches, such as single-cell RNA sequencing, with functional screening of inferred cellular diversity, are starting to reveal microglia diversity. The application of single-cell techniques to phenotype homeostatic and activated microglia under different neurological disorders are currently opening new avenues to understand cellular and functional states of various subpopulations in a context-dependent manner, thus shedding more light on microglia unique features.

## Author Contributions

AM wrote the manuscript. All the authors edited and approved the manuscript.

## Conflict of Interest Statement

The authors declare that the research was conducted in the absence of any commercial or financial relationship that could be construed as a potential conflict of interest.
